# Conceptual Design of a Hybrid Composite to Metal Joint for Naval Vessels Applications

**DOI:** 10.3390/ma18153512

**Published:** 2025-07-26

**Authors:** Man Chi Cheung, Nenad Djordjevic, Chris Worrall, Rade Vignjevic, Mihalis Kazilas, Kevin Hughes

**Affiliations:** 1 Centre for Assessment of Structures and Materials Under Extreme Conditions (CASMEC), Department of Mechanical and Aerospace Engineering, Brunel University London, London UB8 3PH, UK; manchi.cheung@twi.co.uk (M.C.C.);; 2 TWI Ltd., Granta Park, Great Abington, Cambridge CB21 6AL, UK; 3 TWI, Brunel Composite Centre, Granta Park, Cambridge CB21 6AL, UK

**Keywords:** friction stir spot welding, glass fibre fabric, simulation-led design, finite element methods

## Abstract

This paper describes the development of a new hybrid composite for the metal joints of aluminium and glass fibre composite adherents. The aluminium adherend is manufactured using friction stir-formed studs that are inserted into the composite adherend in the through-thickness direction during the composite manufacturing process, where the dry fibres are displaced to accommodate the studs before the resin infusion process. The materials used were AA6082-T6 aluminium and plain-woven E-glass fabric reinforced epoxy, with primary applications in naval vessels. This joining approach offers a cost-effective solution that does not require complicated onsite welding. The joint design was developed based on a simulation test program with finite element analysis, followed by experimental characterisation and validation. The design solution was analysed in terms of the force displacement response, sequence of load transfer, and characterisation of the joint failure modes.

## 1. Introduction

The application of composites in the naval industry overcomes two major problems: corrosion and the weight of the topside/superstructure. Composites also enhance operational performance [1] and reduce ownership costs, including maintenance and fuel consumption [2]. The current application of composites in naval vessels was reviewed in [2]. The trend shows extensive use and promising further applications, primarily driven by the need to reduce acquisition and maintenance costs, while improving operational performance. However, metals still have a significant role in several areas of the vessel structures, due to their machinability, ductility, and toughness, including better resistance to fire, which is why metals are still the material of choice for deck design [3].

Adhesively bonded joints between dissimilar materials, while offering smooth stress distribution and minimal stress concentration, are often limited by brittle failure and a lack of residual strength after debonding. In contrast, mechanically fastened joints provide improved damage tolerance and repairability but introduce additional weight and aesthetic constraints to the structure. Consequently, there is a strong need for robust and reliable composite-to-metal joints to enable effective structural integration.

Composite-to-metal joints have been the subject of several investigations. Examples include the comprehensive simulation and testing program of large-scale bolted and bonded joints connecting composite shells to steel decks in naval vessels [4]. The mechanical performance of a joint between a sandwich glass fibre-based composite superstructure and a steel hull was investigated in [5]. The French Navy implemented this concept on the French La Fayette-class frigates for the connection between a composite helicopter hangar and weather deck [6], where the composite skins were adhesively bonded to the steel.

Previous research has explored a variety of hybrid joint designs, including literature reviews on composite-to-metal joining technologies [7,8,9,10,11]. Generally, there are four types of joining technologies: adhesive bonding, mechanical fastening, welding-based technologies, and hybrid technologies. Among hybrid technologies, the category that is the most relevant for this work is based on the introduction of through-thickness elements in the manufacturing before consolidation of the cured composite laminate, which is known as “through-the-thickness reinforcement” or “direct assembly”. Through-the-thickness reinforcement provides mechanical interlocking between the composite and metal adherends and allows for a greater load to be transferred between the adherents; this type of joint has already been used in the automotive or aircraft industry [9]. Although “through-the-thickness” joining technology has been developed, research has typically focused on the fabrication of metal pins with complex geometries, including a range of shapes and heights [12] or inclination angles [13]. These are typically based on the application of additive manufacturing and are difficult to achieve using conventional manufacturing methods. In addition, the dimensions of these features remain relatively small, with reported diameters ranging from 0.3 mm in [14], 0.5 mm in [13], 1 mm in [12], to 1.6 mm in [15], which makes the designs suitable for high-precision applications such as the aerospace industry, where space and weight constraints are critical, rather than for large composite structures such as naval vessels.

To enable the scalable application of hybrid joining technologies, mechanical reinforcement features with larger dimensions that are compatible with coarse-weave composite fabrics and industry-relevant manufacturing techniques, such as vacuum-assisted resin infusion, are required. Moreover, there is a lack of experimental data investigating the mechanical interactions between a single feature and an adhesive surface in composite-metal joints. Existing studies typically focus on multi-pin arrangements, leaving the fundamental behaviour of a single reinforcement element underexplored. This study addresses this gap by introducing a displacive method to form embedded metal studs that do not increase the overall structural weight of the joint compared to the equivalent reference adhesive joint. The hybrid joint was manufactured via vacuum resin infusion, ensuring compatibility with large-scale composite fabrication processes. One possible solution is to develop a composite-to-metal joint for a composite superstructure with a metallic edge that would interface with the rest of the metallic vessel structure. This interface is meant to be easily welded using conventional shipyard welding techniques, including arc welding. This joining approach offers a cost-effective solution that does not require complicated onsite welding.

Consequently, the aim of the project presented here is to develop and characterise a novel composite-to-metal joint, which consists of an aluminium adherent with a friction-formed stud as the through-thickness connector and a glass fibre composite adherent. The materials used in this study were AA6082-T6 aluminium and plain-woven E-glass fabric-reinforced epoxy. This work investigates the structural performance of a new single-lap composite-to-metal joint reinforced with embedded metal studs (MSJ) and compares it with that of reference adhesive joints without reinforcement (RIJ). The comparative evaluation includes the following:

- Load-bearing capacity,
- Energy absorption before and after peak load
- Joint stiffness,
- Failure mechanisms, and
- Influence of the overlap length.

These findings provide valuable insights into the post-failure behaviour, load transfer mechanisms, and damage evolution in hybrid joints reinforced by a single embedded metal feature. The results offer a fundamental understanding of the behaviour and load-transfer mechanism in such joint configurations and support the design and optimisation of future multi-stud hybrid joints to meet the demands of naval and other industries.

## 2. Methodology

The methodology developed in this work consists of the conceptual design of a new joint, a simulation program to support the design, and an experimental program to validate the design, which are described in the following subsections.

### 2.1. Conceptual Design

Technological advancements are driven by practical industrial needs, starting from the design requirements. The proposed design aims to enhance the efficiency, productivity, and competitiveness of the new joint in industrial settings; therefore, the specific design requirements are as follows:

- Material compatibility with marine environments
- The joint design should satisfy the lightweight requirements—minimising weight and avoiding the use of mechanical fasteners, which will also satisfy the aesthetics requirements.
- The principal load is shear, originating from the composite superstructure weight.
- Strength requirements: The novel design must not be overperformed by the available alternatives.
- Joint manufacturing is suitable for naval vessels.
- Compliance with standards: The joint design should comply with relevant industry standards, naval specifications, and regulatory requirements.

The proposed design solution is to modify the surface of the metal component to include stud features, which are embedded into the composite adherent in the through-thickness direction, so that the joint combines the mechanical interlock and adhesive bonding strength of the composite matrix material. To produce novel composite-to-metal stud joints, AA6082, a widely used marine-grade aluminium alloy [16], and plain-woven E-glass fabric reinforced epoxy were chosen as the adherend materials.

**Figure 1:**
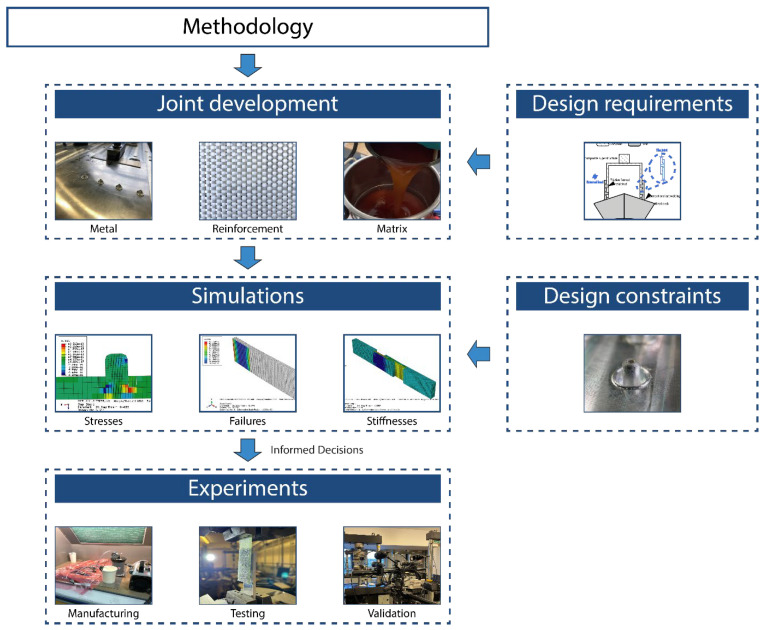
Methodology developed for the simulation-led design.

The simulation-based methodology developed for the conceptual design is illustrated in Figure 1. Starting from the design requirements and material selection specified above, the process started from the representative sample selection and simulation program, with the aim of determining the required maximum strength and other design requirements, and proceeded towards the prototype demonstrator.

**Figure 2:**
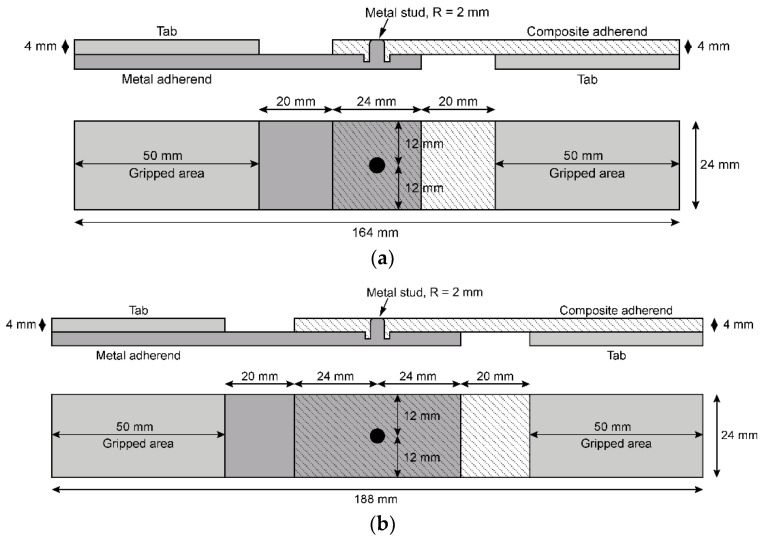
Novel hybrid joint solutions: (**a**) hybrid joint with a standard bonding area, denoted as MSJ-L24; and (**b**) hybrid joint with double the size of the bonding area, denoted as MSJ-L48.

Currently, no standards are available for composite-to-metal joints with through-thickness metallic features. Consequently, two specimen types were considered: (a) the specimens defined in the ASTM D5961/D5961M-17 Standard Test Method for Bearing Response of Polymer Matrix Composite Laminates [17], denoted as MSJ-L24, and (b) the specimen with double the size of the bonding area, denoted as MSJ-L48. The specimens are illustrated in Figure 2. The metal stud was introduced at the centre of the bonding area, as this location corresponded to the region of minimum stress under shear loading conditions. Details of the geometry, including the edge distance to hole diameter (e/d) and specimen width to hole diameter (w/d) ratios, are provided in Table 1. Given the thickness of the metallic adherent, the maximum stud diameter was 4 mm, and the minimum specimen width was 24 mm, which implied a minimum edge of 12 mm for specimen type MSJ-L24 and 24 mm for specimen type MSJ-L48.

The novel joints were compared to reference adhesively bonded joints with the same size of the bonding area.

### 2.2. Finite Element Models Development

The primary objective of the simulation framework was to make informed decisions for the design of the experiment in terms of assessing the integrity of the joint design, including stress distribution, failure sequence, and integrity of the stud and composite at the maximum load. All simulations were performed using Abaqus CAE 2020 [18].

**Figure 3:**
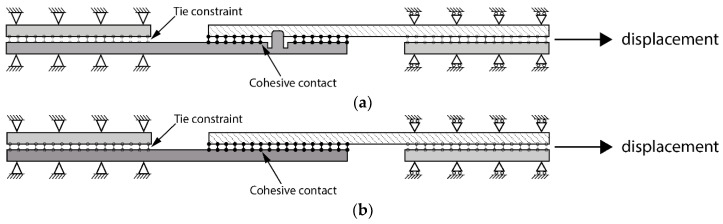
Joint configuration loading and boundary conditions: (**a**) new joint and (**b**) reference adhesively bonded joint.

Two modelling approaches were used in this work: models developed with the thick shell element formulation and models developed using linear hexahedral elements. The key difference between the models was the modelling of failure in the composite material: the former used the Hashin failure criterion available in Abaqus [18], while the latter used a user material subroutine with Hashin criteria for solid elements, as described below. The global element size was 0.5 mm, which is equivalent to eight elements through the thickness of the adherent, where each ply of the composite was modelled separately. The loading and boundary conditions for the reference and new joint configurations are shown in Figure 3. The cohesive contact was used for the bonding overlap area, with the tied contact assigned between the adherents and tabs at the end of the specimens. No sliding between the tabs and the adherents was anticipated. One end of the joint was fully fixed, while a longitudinal displacement was applied at the other end, as illustrated in the figure.

In the solid FEM models, the composite material was modelled with orthotropic elastic material properties and the Hashin failure criterion, which was developed and implemented as a user material subroutine (UMAT) for hexahedral solid elements in Abaqus [18]. The Hashin damage initiation criterion considers each failure mode separately, using the following set of equations for fibre and matrix failure, respectively [19]:

(1) $${\left(\frac{\sigma_{11}}{X_{T}}\right)}^{2}+\left(\frac{{\sigma_{12}}^{2}+{\sigma_{13}}^{2}}{{S_{12}}^{2}}\right)\geq 1\;\mathrm{for}\;\sigma_{11}\geq 0$$

(2) $${\left(\frac{\sigma_{11}}{X_{C}}\right)}^{2}\geq 1\;\mathrm{for}\;\sigma_{11}<0$$

(3) $${\left(\frac{\sigma_{22}+\sigma_{33}}{{Y_{T}}^{2}}\right)}^{2}+\frac{{\sigma_{23}}^{2}-\sigma_{22}\sigma_{33}}{{S_{23}}^{2}}+\frac{{\sigma_{12}}^{2}+{\sigma_{13}}^{2}}{{S_{12}}^{2}}\geq 1\;\mathrm{for}\;\;\sigma_{22}+\sigma_{33}\geq 0$$

(4) $$\left[{\left(\frac{Y_{C}}{2S_{23}}\right)}^{2}-1\right]\left(\frac{\sigma_{22}+\sigma_{33}}{Y_{C}}\right)+{\left(\frac{\sigma_{22}+\sigma_{33}}{4{S_{23}}^{2}}\right)}^{2}+\frac{{\sigma_{23}}^{2}-\sigma_{22}\sigma_{33}}{{S_{23}}^{2}}+\frac{{\sigma_{12}}^{2}+{\sigma_{13}}^{2}}{{S_{12}}^{2}}\geq 1\;\sigma_{22}+\sigma_{33}<0$$

where $X_{T}$ and $X_{C}$ represent laminate strength in tension and compression in principle material direction X, respectively, $Y_{T}$ and $Y_{C}$ laminate strength in principle material direction Y and $S_{12}$ and $S_{23}$ are the laminate shear strength in the principle material planes. The expressions on the left are postprocessed as state variables SDV1 to SDV4 in Abaqus.

**Figure 4:**
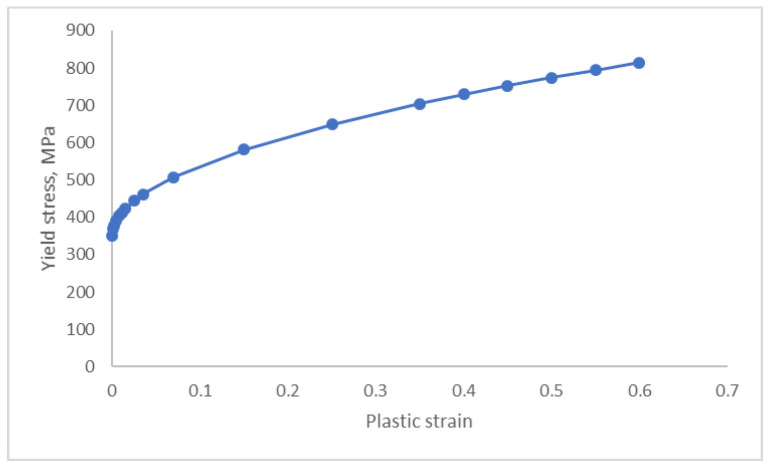
Inelastic part of the material response assigned to the aluminium adherent.

The composite material fails when the expressions on the left exceed 1.0. The current version of the model assumes brittle behaviour and complete loss of strength at the failure point. The material parameters for the composite material were obtained from [14]. Aluminium was modelled as a rate-independent isotropic elastic-plastic material with a Young’s modulus of 73,800 MPa, Poisson’s ratio of 0.33, and density of 2.7 × 10^−9^ tonne/mm^3^. The inelastic part of the material response was assigned using the stress—plastic strain relationship shown in Figure 4.

**Figure 5:**
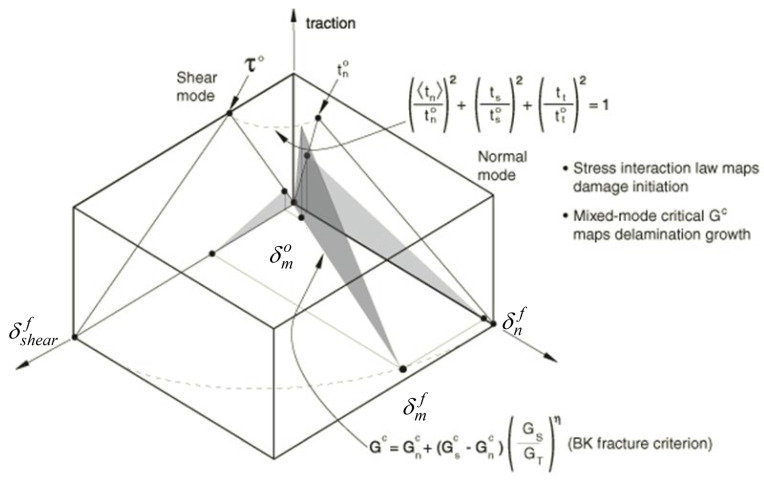
Benzeggagh-Kenane fracture criterion for mixed-loading mode [18].

Cohesive contact was modelled using the bilinear traction-separation law, illustrated in Figure 5, with the Benzeggagh-Kenane fracture criterion for debonding propagation for mixed-mode (I, II, and III) [18]. The uncoupled contact stiffness coefficients were 85.1 MPa in the normal direction and 25.76 MPa in the shear direction relative to the overlapped surfaces [20]. The area under the traction-separation curve represents the fracture energy [20,21]. The material properties used in the simulations are given in [15].

### 2.3. Experimental Programme

The experimental program included manufacturing, testing, and validation against the simulation results. To demonstrate the benefits of the newly developed joint, its performance was compared with that of a reference adhesively bonded joint. The effects of the size of the bonding—overlap area were also investigated, and two configurations were manufactured: one with a squared bonding area equivalent to the standard and denoted as MSJ-L24 [17], and one with double the size of the standard bonding area, MSJ-L48.

#### Manufacturing of the Samples

**Figure 6:**
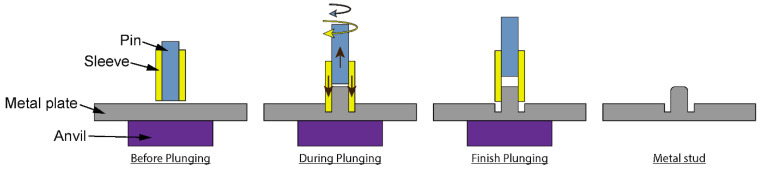
Metal stud manufacturing process with the arrows illustrating motion of the RFSSW tool [22].

**Figure 7:**
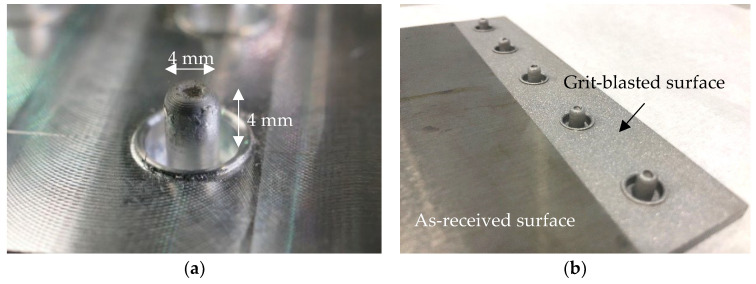
(**a**) Metal stud as manufactured and (**b**) surface treatment in the bonding area.

The first stage of manufacturing is the formation of metal studs using a refill friction stir spot welding (RFSSW) tool, following the process illustrated in Figure 6. Contact between the metal plate and the sleeve produces frictional heat, which softens the base material and allows for the flow and restructuring of the metal plate. At the same time, vertical motion of the rotating pin creates a cavity and drags the softened material upwards. As a result, a stud is formed on the surface of the metal plate. The kinematics of the tool controls the geometry of the stud, with the dimensions of the metal stud formed in this work shown in Figure 7a; the cavity depth was 2 mm. The manufacturing of the studs was repetitive, with the stud-to-plunge depth being the most varying parameter, which was controlled by the RFSSW tool. Following the manufacturing of the studs, the bonding area of the aluminium plates was treated by using grit blasting so that the plates at the end of the process are shown in Figure 7b. The last step in processing the aluminium adherents was the removal of the ring around the metal stud to avoid potential stress concentration in the composite adherent and improve the quality of the bonding.

**Figure 8:**
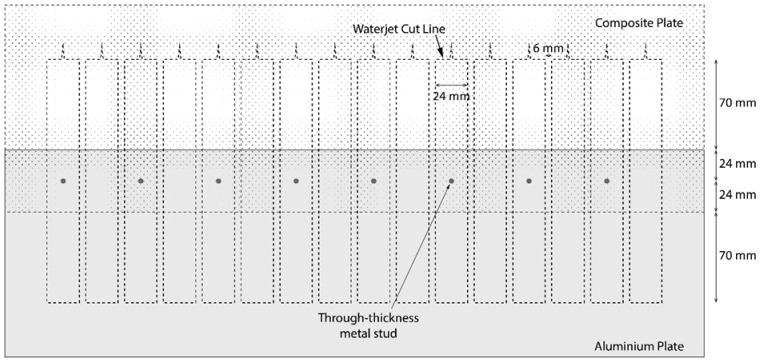
Specimen manufacturing plan.

**Figure 9:**
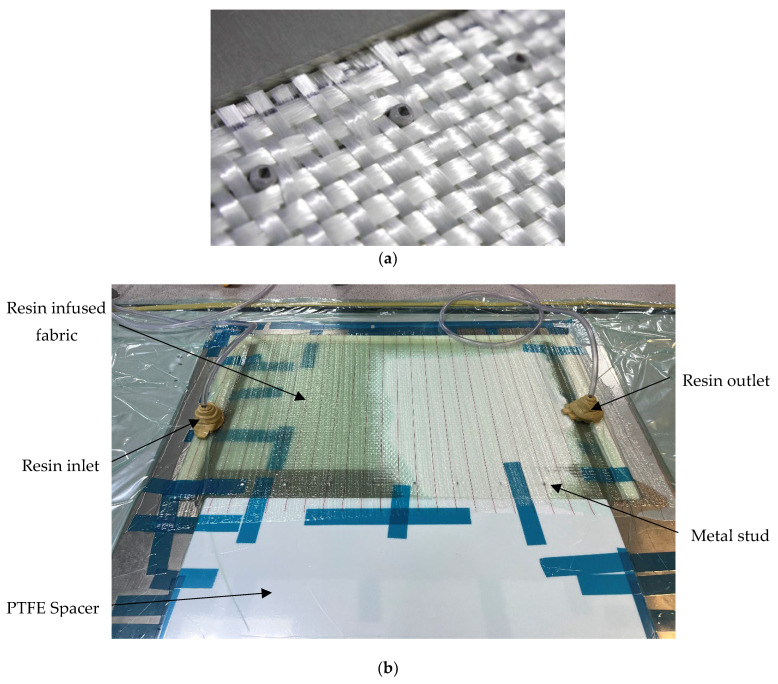
Sample manufacturing: (**a**) dry fabric and (**b**) vacuum-assisted resin infusion.

**Figure 10:**
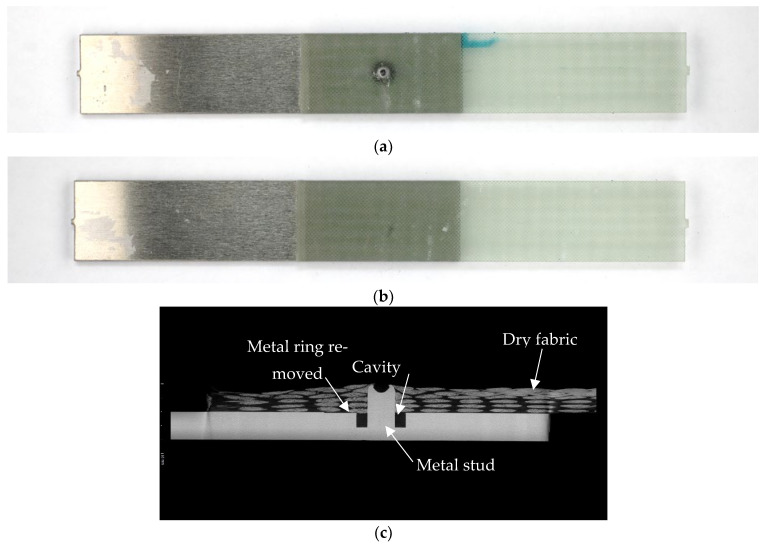
(**a**) MSJ-L48 specimen of the new joint (with metal); (**b**) reference RIJ-L48 adhesively bonded joint; and (**c**) XCT image of the overlap area of the MSJ-L48sample.

To minimise the uncertainties related to manufacturing, specimens with the same size of the bonding area were cut from the assembly manufactured in a single operation, following the plan illustrated in Figure 8. Consequently, metal studs were formed on an AA6082-T6 aluminium plate with dimensions of 540 mm × 160 mm × 4 mm and 30 mm separation to accommodate the specimens, as illustrated in Figure 8. The joint was then manufactured using the vacuum-assisted resin infusion process. The first step was to lay out five layers of dry fabric, up to the required thickness of the composite adherent, so that the fibres were aligned with the longitudinal—axial specimen direction, as shown in Figure 9a. This was followed by resin infusion in the setup shown in Figure 9b to form the composite adherent. The vacuum bag is connected to a vacuum pump via the resin outlet to remove air from the mould cavity. As air is removed, the vacuum pressure draws the resin into the dry fibre stack. The infused composite laminate is left under vacuum until the resin is fully cured and hardened. The resin infusion process was conducted at room temperature for 24 h for curing and for another 24 h under the same conditions for post-curing. The last step in sample manufacturing was waterjet cutting. The MSJ-L48 and RIJ-L48 specimens are shown in Figure 10a,b.

Due to the manufacturing process, the quality of the specimens had to be inspected, but that task was challenging due to the adherent material dissimilarity and the resin infusion manufacturing process. Consequently, a joint inspection was carried out with a selection of the samples using X-Ray Computed Tomography (XCT) before the specimens were tested. The quality of the XCT-inspected joints was satisfactory, as illustrated in an example given in Figure 10c.

## 3. Results and Discussion

### 3.1. Simulation Results

**Figure 11:**
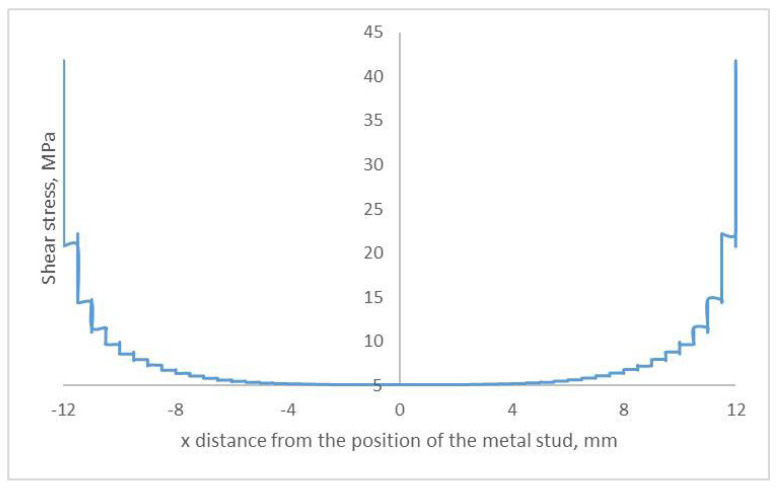
Longitudinal stress distribution along the length of the reference adhesively bonded joint.

The simulation results for the in-plane longitudinal stress distribution along the length of the reference adhesively bonded joint are shown in Figure 11. The maximum stress of 39 MPa was obtained at the edge of the overlapped area, with the minimum stress at the centre of the overlapped area close to zero. This result agrees well with the analytical result obtained using the Goland and Reissner equations, which confirms that the model represents the physical system appropriately.

**Figure 12:**
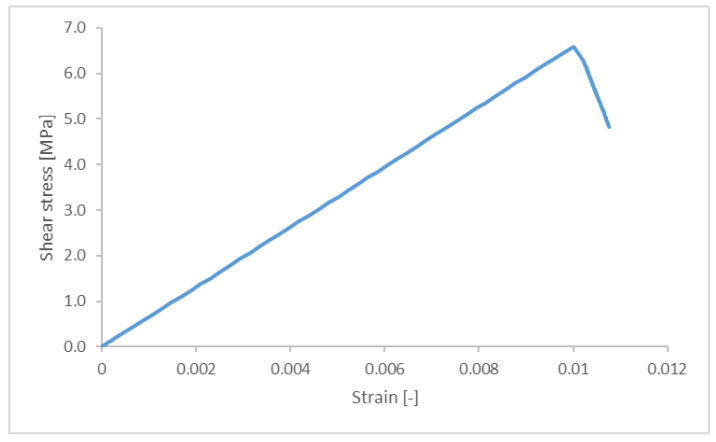
Stress–strain curves obtained in the simulation of the reference adhesively bonded joint.

The simulation results for the force-displacement curves of the adhesively bonded joint were obtained using the solid model and based on the results at the node where the loading was applied. The slope of the curve was constant, equal to 15,830 N/mm, up to a maximum of 7.28 kN. The sharp unloading was a consequence of the bonding failure with the last calculated step at a force of 3.73 kN and a displacement of 0.53 mm. This result was converted to the stress–strain relationship shown in Figure 12, with the maximum stress equal to 6.32 MPa.

**Figure 13:**
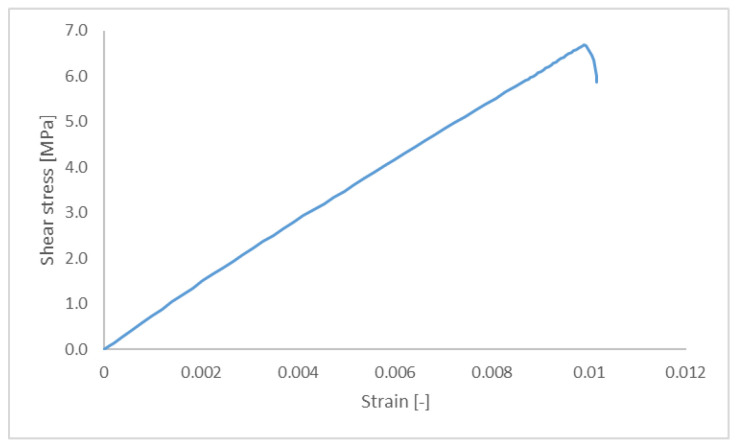
Stress–strain curves obtained in the simulation of the novel hybrid joint.

Equivalent simulation results obtained with the FEM solid model of the novel joint were used to calculate the force-displacement and stress−strain curves. The constant force-displacement curve gradient was 15.47 kN/mm from the beginning of the loading to a maximum of 7.06 kN. The stress−strain curve obtained using the novel joint is shown in Figure 13. The maximum load also corresponded to complete failure in the bonding area. However, the main deficiency of the simulation programme was that the simulation terminated before complete joint failure. The results obtained in the simulation programme suggest that the deformation process of the new joint follows the behaviour of the reference adhesively bonded joint up to the complete debonding of the adherents, which means that the bonding stiffness and its strength control this phase of the deformation process.

**Figure 14:**
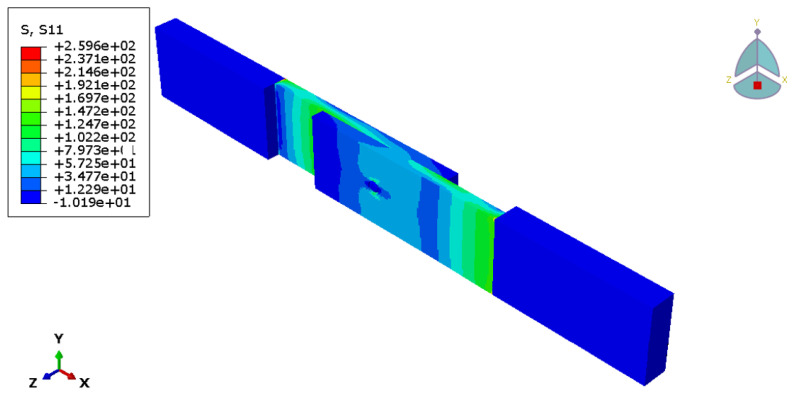
Stress distribution in the novel hybrid joint model.

**Figure 15:**
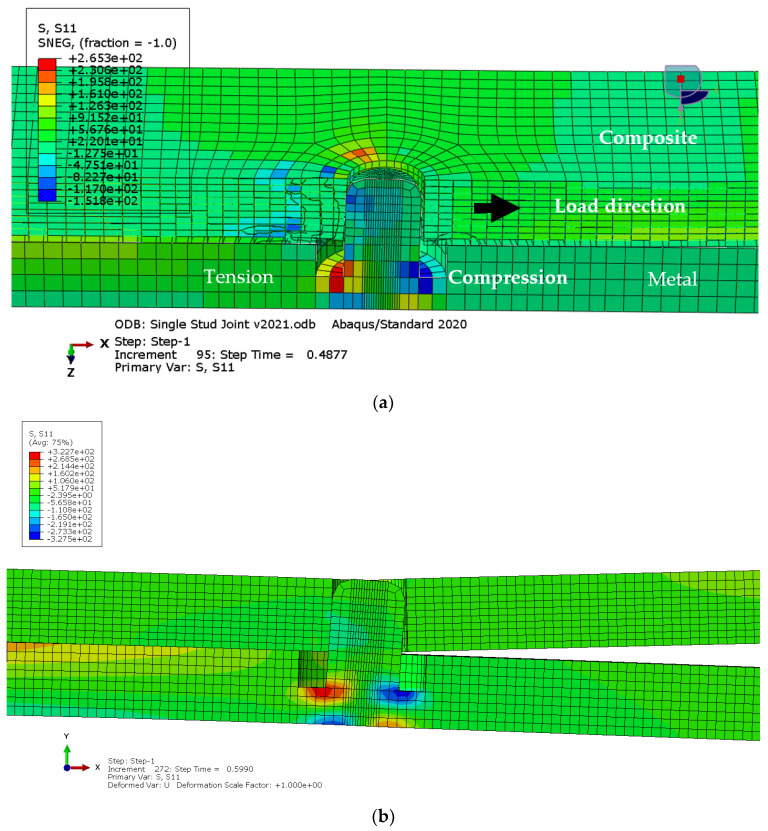
Stress distribution in the vicinity of stud in the novel hybrid joint model: (**a**) thick shell model; (**b**) solid model.

Figure 14 shows stress distribution in the longitudinal direction, with the extreme values obtained in the vicinity of the edges of the bonding area and around the stud due to stress concentration. The simulation captured the bending of the stud, as shown in Figure 15, with the maximum magnitude of the principal stress obtained in the stud root and on the free surface of the metal adherent. The stress levels are very close to the material yield stress, which suggests that plastic deformation is expected in this area of the metal adherent.

**Figure 16:**
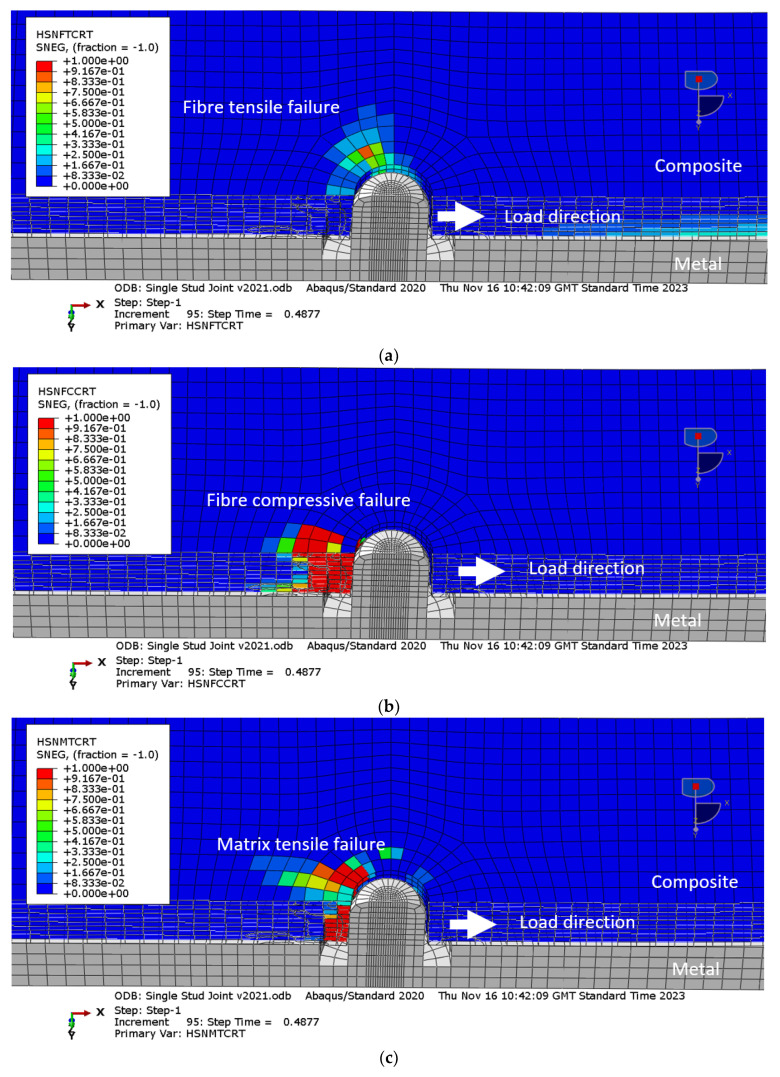

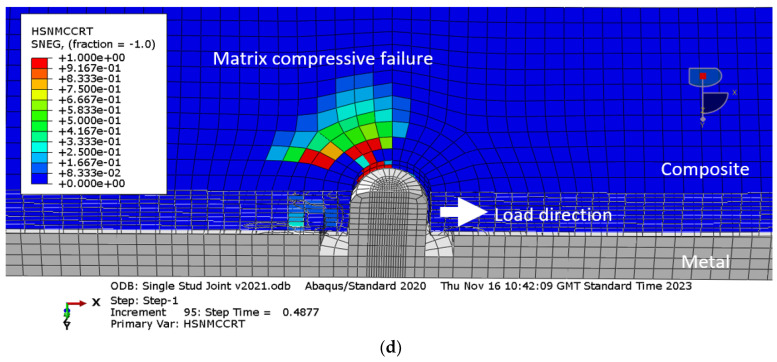
Hashin failure criteria obtained with the thick shell element model at the last calculated step: (**a**) fibre tensile failure; (**b**) fibre compression failure; (**c**) matrix tensile failure; (d) matrix compression failure.

**Figure 17:**
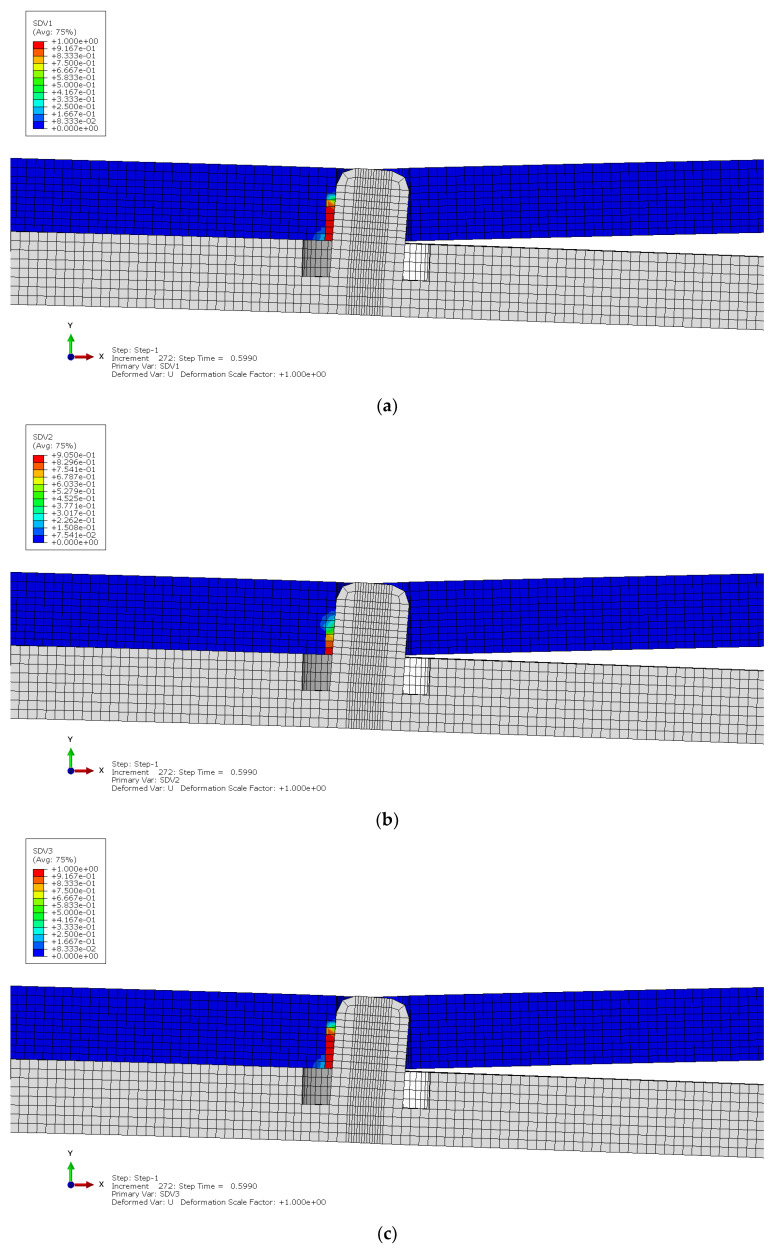

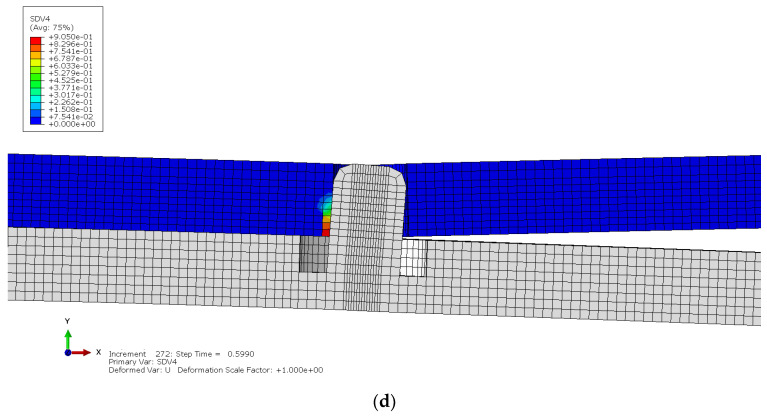
Hashin failure criteria obtained with the solid element model at the last calculated step: (**a**) fibre tensile failure (SDV1); (**b**) fibre compression failure (SDV2); (**c**) matrix tensile failure (SDV3); (**d**) matrix compression failure (SDV4).

Simulation results for the composite adherent failure, obtained with Hashin’s failure criteria with thick shell elements model and the solid element model, are shown in Figure 16 and Figure 17, respectively. Hashin’s failure criteria calculate the failure indices, which are between 0.0 and 1.0 for the virgin and failed material, respectively. Although the simulations stopped shortly after the bonding failure, both results predicted fibre failure and matrix failure in the composite material in the vicinity of the stud, with the solid element models slightly underestimating the extent of damage. More specifically, the fibre tensile failure was obtained due to stress concentration developed locally near the stud, see Figure 16a, whilst the compressive failure was induced due to contact between the composite and the stud in the direction of the applied load, see Figure 16b. Similar behaviour was observed with the matrix failure, Figure 16c,d. Importantly, the volume of the failed material is not significant, so some residual strength of the joint post bonding failure was expected. However, the key limitation of the simulation results was that post-bonding failure behaviour was not obtained, due to limitations of the numerical tools used.

### 3.2. Experimental Results

Performance of the joint was compared to a reference adhesively bonded joint in terms of the following criteria:

- Load-bearing capacity defined as the maximum force sustained before failure, as a critical property;
- Load-displacement curve and its main features, including yielding and post maximum behaviour;
- Fracture energy required for opening a new surface, determined by the area under the load-displacement curves;
- Failure modes in terms of dominant damage mechanisms;
- Stiffness, determined as a slope of the stress−strain curve in the elastic region, between strains of 0.05% and 0.25%;
- Stress distribution and stress concentrations, as a potential location for crack initiation or failure;
- Failure in the bonding area, determined by relative displacement in the out-of-plane direction of the two adherends;
- Sliding distance, as a relative axial displacement between the adherents, determined by the relative displacement of the two adherends.

In addition, although the specimens were cut from the same assembly and precisely to the same geometry, the test results can vary, which is why a statistical analysis of the variability due to manufacturing and testing inconsistencies was carried out. This was achieved by calculating statistical metrics, such as mean, standard deviation, and confidence intervals. For all tests conducted, a coefficient of variation (COV) below 10% was considered acceptable/consistent. The analysis also included a detection and treatment of the outliers, following the ISO 16269 Statistical interpretation of data—Part 4 [23]. For any detected outlier, the value is either corrected or discarded based on the identifiable cause of the error, such as clerical error, dilution error, measurement error, etc. If the presence of the outliers cannot be reasonably explained, the outliers were treated as valid observations. Analyses with and without the outliers were conducted to identify the influence of outlying observations on the results of data analysis without deleting them as suggested by ISO 16269.

#### 3.2.1. Force Displacement Response

**Figure 18:**
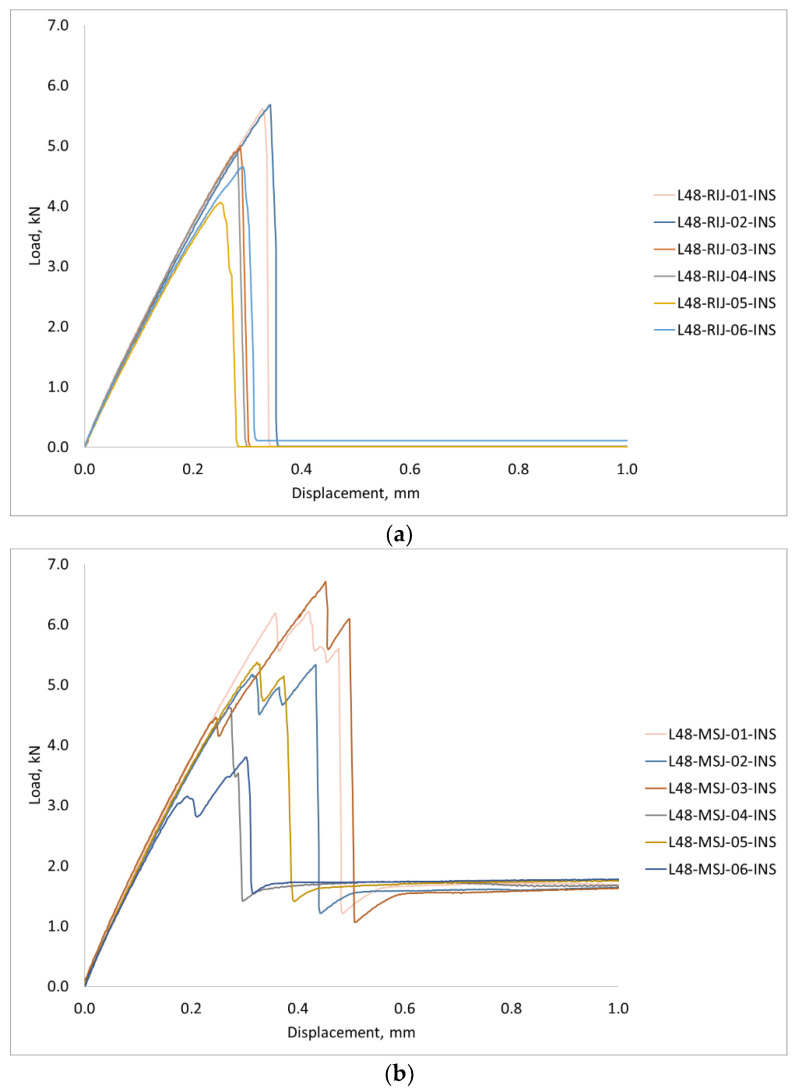
Load Displacement obtained with the 48 mm overlap length: (**a**) reference adhesively bonded joint (L48-RIJ); (**b**) novel metal stud joint (L48-MSJ).

The first set of tests was conducted with six specimens with metal studs and six reference specimens, with double the size of the bonding area relative to the standard single lap specimen, which are denoted as MSJ-L48 and RIJ-L48 specimens, respectively. The experimental results for the force displacement curves are shown in Figure 18. Both curves show almost linear behaviour up to the maximum force, but the post-peak behaviour is significantly different.

The reference adhesively bonded joint had the maximum load between 5.68 kN and 4.07 kN, which corresponds to a mean of 4.98 kN and a standard deviation of 0.61 kN. The mean displacement at the maximum force for these tests was 0.31 mm. In all tests, the joint suddenly failed by debonding of the adherents and load drop to zero.

**Figure 19:**
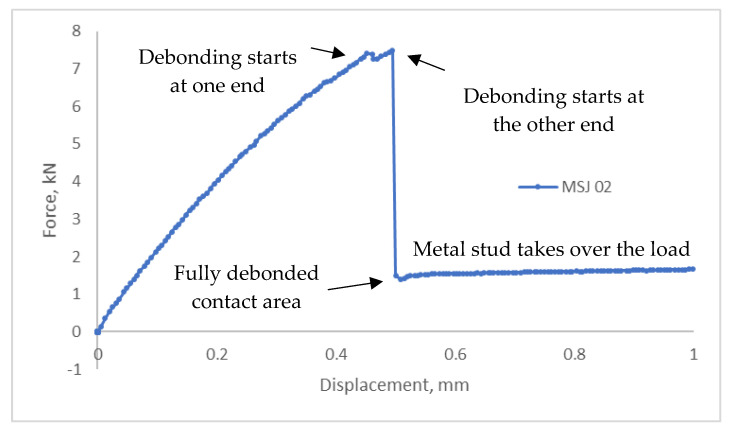
Typical force-displacement curve recorded with the new metal stud hybrid joint and the characteristic response.

**Figure 20:**
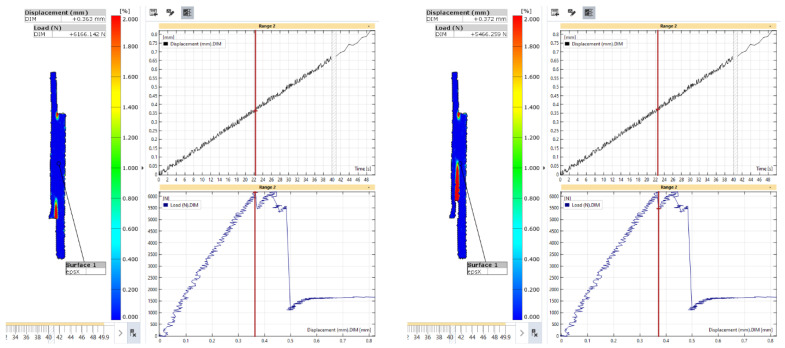
DIC data—Strain field in the novel metal-stud joint.

**Figure A1:**
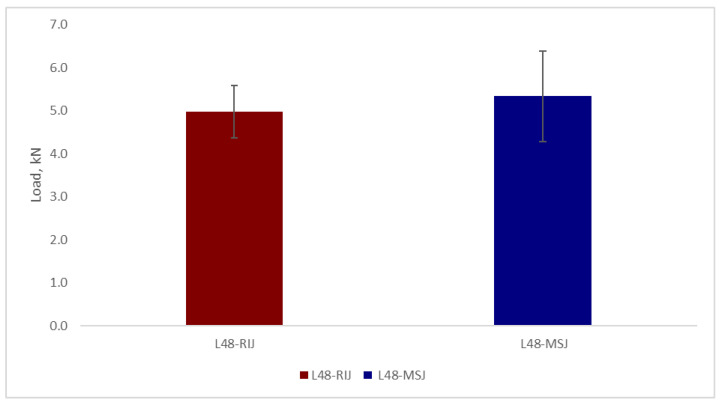
Comparison of the mean maximum loads obtained with the reference L48-RIJ and new L48-MSJ joints.

The load-displacement curve for L48-MSJ showed a similar initial behaviour as the reference joint, see Figure 18b, and with the maximum force recorded to be between 6.71 kN and 3.80 kN, which corresponds to the mean and standard deviation of 5.34 kN and 1.05 KN, respectively. Comparison between this result and the reference result is given in Figure A1 in the Appendix A. The mean displacement at the peak was 0.37 mm. The force displacement curves recorded in the six tests showed consistent behaviour, with several characteristic points shown in Figure 19, which were observed based on the digital image correlation (DIC) data, see Figure 20. The first change in the curve gradient was observed when debonding between the adherents was initiated on one side, which was followed by bonding failure on the other side of the debonding area (second peak). Bonding failure then propagated inwards up to the first minimum force, as shown in Figure 19. A loading of around 1.9 kN was then solely transferred and resisted by the coupling between the stud and the composite when the deformation process featured bending of the stud and bearing failure in the composite adherent. A detailed analysis of the axial strain state is provided in Section 3.2.3.

In terms of the fracture energy, calculated as the area under the curves of the two sets of L48 tests, MSJ-L48 had a mean fracture energy of 2.35 J with a standard deviation of 0.37 J, which was over two times greater than the mean fracture energy of 0.88 J recorded for the RIJ -L48 specimens with a standard deviation of 0.17 J. The results showed that the metal stud significantly enhanced the energy absorption capacity compared to the reference joints and effectively contributed to delaying the complete separation of the adherents.

**Figure 21:**
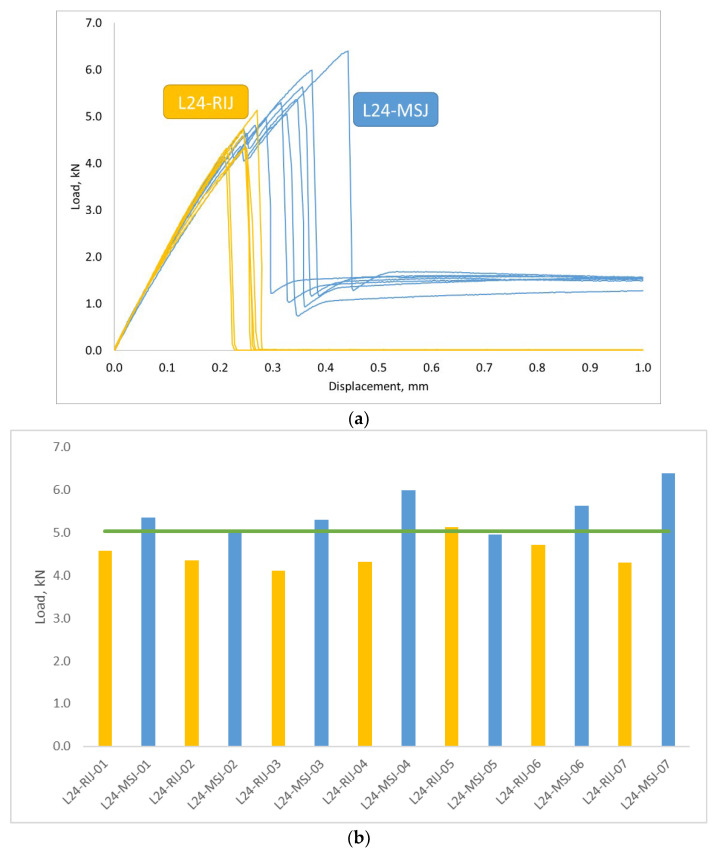
(**a**) Load Displacement curves obtained with a 24 mm overlap length. (**b**) Comparison of the maximum loads obtained in the two tests. Green line represent average.

An equivalent test program was also carried out with the standard-size specimens [17], denoted as L24-RIJ and L24-MSJ for the reference and new metal stud joints, respectively. The results of these tests are shown in Figure 21, with very good repeatability and the same features observed in the L48 tests.

**Figure 22:**
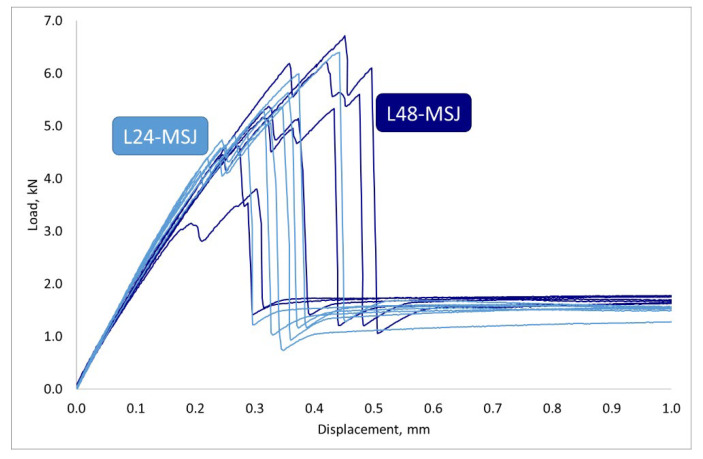
Force displacement curves obtained with the 24 mm (light blue L24-MSJ) and 48 mm long bonding areas (navy blue L48-MSJ).

A comparison of the results obtained for the novel joints with two sizes of the bonding area is shown in Figure 22. It can be observed that an increase in the size of the bonding area did not significantly contribute to the dissipated energy in the debonding process and did not affect the post-peak behaviour of the joint.

#### 3.2.2. Post Failure and Fracture Surface Analysis

**Figure 23:**
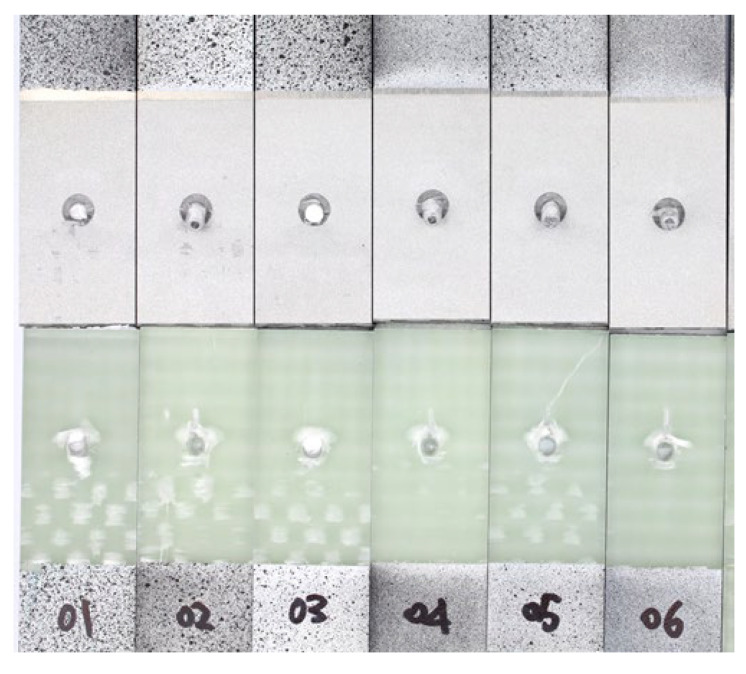
Fracture surface of all L48-MSJ specimens.

Figure 23 shows the fracture surfaces of all the L48-MSJ specimens. The region exhibiting bearing failure caused by the metal stud was similar in size across the six specimens. The metal stud underwent fracture in two tests, L48-MSJ-01 and L48-MSJ-03, which demonstrated the highest load-bearing capacity, as shown in Figure 18b. The studs in the other four tests underwent bending and pronounced plastic deformation. L48-MSJ-04 and L48-MSJ-06 did not show visible damage to the composite adherend, which may be explained by the quality of bonding and load transfer at the joint interface.

The stud failure modes in the L48-MSJ-01 and L48-MSJ-03 tests were distinctly different from each other: Specimen L48-MSJ-01 exhibited ductile failure with significant plastic deformation prior to failure, which suggests a greater energy absorption before complete joint failure. In contrast, specimen L48-MSJ-03 exhibited signs of brittle stud failure without pronounced plastic deformation before failure. These failure modes may be due to imperfections in the manufactured studs and require further investigation. Ductile failure is more damage tolerant and is consequently the preferred failure scenario.

**Figure 24:**
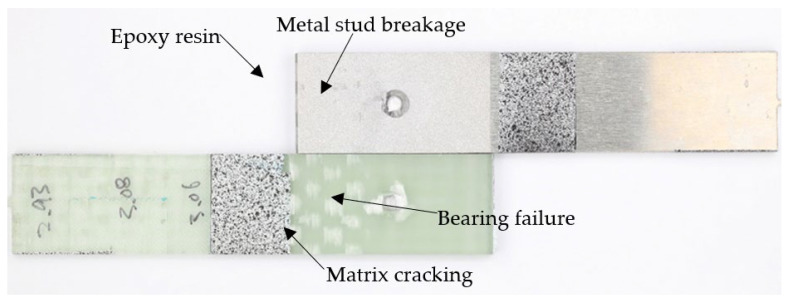
Fracture surface of L48-MSJ-01 with metal-stud breakage.

Figure 24 shows the fracture surface of specimen L48-MSJ-01, with several distinct failure features, including metal stud fracture. The fracture occurred in the bonding area plane, and after the bonding failure, the load was increasingly transferred to the metal stud. When the applied load exceeded the ultimate strength of the metal stud, it fractured, likely in shear. Notably, traces of epoxy resin were observed on the metal adherent, which confirms the strength of the bonding interface between the adherents.

Localised bearing failure near the metal stud was observed in the composite adherend, which is typical for mechanically fastened joints, where the local compressive stress exceeds the bearing strength of the composite. Matrix cracking close to the metal end was also observed in the pattern of the woven weft. This composite failure was likely due to secondary bending of the composite adherend.

**Figure 25:**
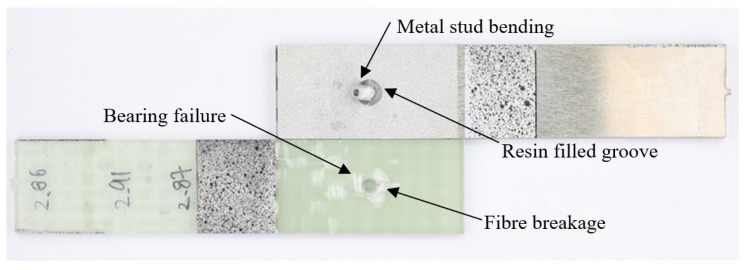
Fracture surface of L48-MSJ-02 without metal-stud breakage.

Figure 25 shows the fracture surface of specimen L48-MSJ-02, where the metal stud exhibited noticeable bending and plastic deformation. Once debonding occurred, the metal stud lost support from the surrounding composite and underwent bending due to the asymmetric loading in the single lap joint. Bearing failure and fibre breakage were observed in the compressed region of the composite adherend. This occurs when the load in the contact zone exceeds the compressive strength of the fibres or the shear strength of the matrix-fibre interface.

#### 3.2.3. Analysis of the Axial Strain for Novel Metal Stud Joint

**Figure 26:**
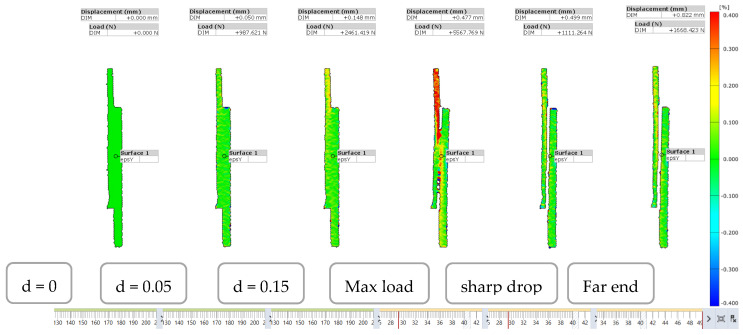
Strain in the axial direction of specimen L48-MSJ-01 at various displacement stages captured from the side.

The distribution of the axial strain in the hybrid L48-MSJ-01 joint, shown in Figure 26 was similar to that obtained with the reference L48-RIJ-01 up to debonding. The axial strain in the composite adherend was higher than that in the metal adherend.

**Figure 27:**
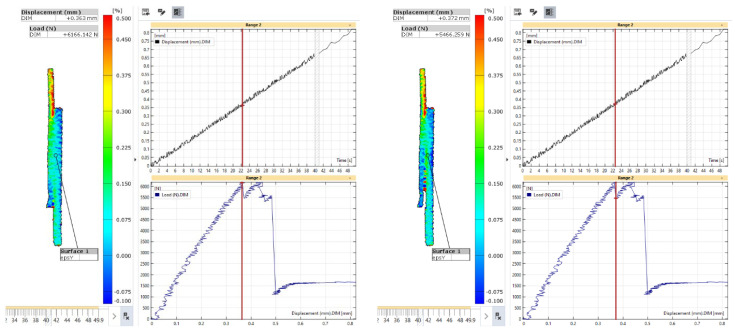
Strain in the axial direction of specimen L48-MSJ-01 at the first local maximum immediately before (**left**) and after (**right**) the sharp drop in load.

**Figure 28:**
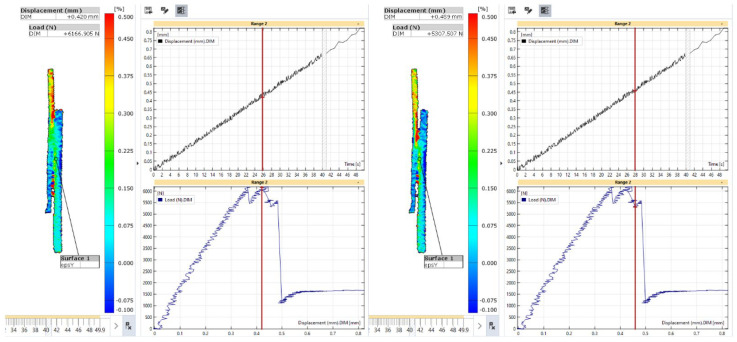
Strain in the axial direction in the L48-MSJ-01 specimen at the second local maximum just before (**left**) and just after (**right**) the sharp drop in load.

To understand the debonding process, the state of the axial strain is presented in Figure 27 and Figure 28 for the first and second load maxima, respectively. For the first maximum in the load-displacement curve, the axial strain showed limited variation immediately before and after the sharp drop in load in the composite adherend. This indicates that the onset of debonding did not significantly affect the overall axial strain distribution across the joint at that time.

At the second maximum load, a high axial strain zone in the composite adherend propagated toward the crack front, as shown in Figure 28. This progression indicates that the load was redistributed and concentrated at the debonding front, leading to the bending of the composite adherend with a reduction in the size of the bonding area. A negative axial strain was developed in the metal adherend at the outer surface of the overlapped area, indicating that the metal adherend also bent away from the composite adherend. Despite these local bending effects at both ends, at this stage of the deformation process, the central part of the bonding area remained intact, providing resistance to the applied load. The localised axial strain near the crack front in the composite adherend was characteristic of damage accumulation and possible failure.

**Figure 29:**
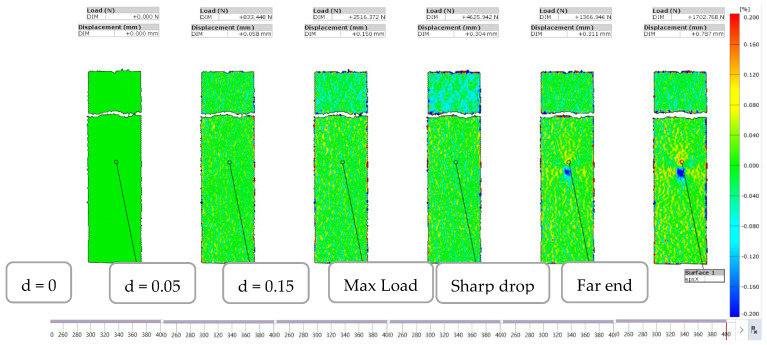
Strain in the lateral direction of specimen L48-MSJ-04 at stages between the maximum load and the immediate sharp drop in load captured from the metal side.

Figure 29 shows that no significant lateral strain concentration was observed on the metal side until the maximum load. However, the lateral strain concentration began to develop following the sharp drop in load, suggesting that the metal stud started to bend after debonding between the adherends occurred.

### 3.3. Comparison of the Simulation and Experimental Results

**Figure 30:**
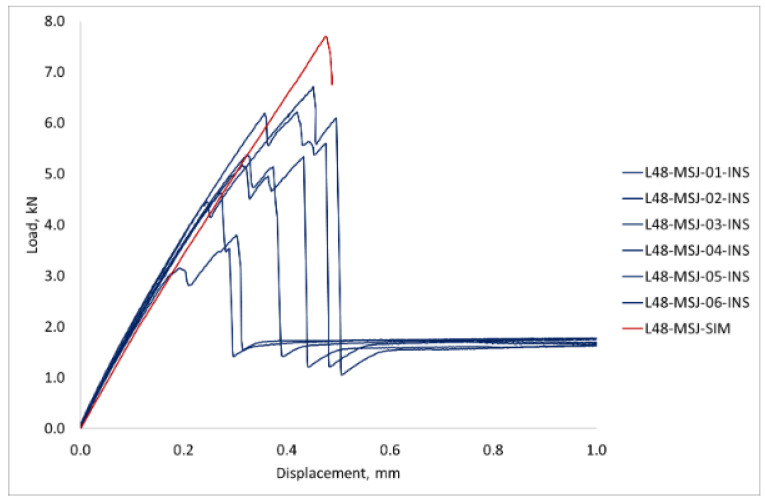
Comparison of the experimental and simulation results obtained for the L48-MSJ specimens.

The simulation results provided valuable insights into the stress distribution and failure mechanisms within the metal stud joint and aided in the development of the experimental program and interpretation of the experimental results. Figure 30 shows the load-displacement curves obtained from the simulation and experiments with the L48-MSJ specimens, where the simulation results for the stiffness of the joint and the displacement at the maximum load-bearing capacity agree well with the experiment. The maximum load was overestimated, which could be a consequence of the material properties used in the simulation for the bonding zone or manufacturing defects.

The simulation results also correctly predicted that debonding at the adhesive interface would be the initial failure mode of the joint under axial loading. This insight was supported by experimental observations from the Digital Image Correlation (DIC) results. The DIC images, taken from the side, showed that debonding initiation and propagation agreed well with the simulation, with the stress concentration starting near the ends of the bonding area. The simulation also helped identify the regions of high shear and peel stress that initiated debonding. This early debonding influenced the subsequent failure evolution, including load redistribution via the metal stud and contribution to the overall failure process. The simulation results also revealed stress concentration due to bending at the root of the metal stud and on the opposite adherent’s free surface, which was observed in the DIC. The stress levels were above the yield stress of the material, which was confirmed by the plastic deformation observed on the free surface of the metallic adherent. It was also observed from both the simulation and experimental results that the composite adherend was undergoing local bearing failure. The simulation suggested that the region where the metal stud was in contact with the composite experienced a localised stress concentration, which caused both matrix and fibre failure. This was confirmed by the analysis of the fracture surfaces of all the specimens.

## 4. Conclusions

This paper presents a new hybrid joint between composite and metallic (aluminium) adherents, where the joint strength combines a large bonding area between the adherents and the contribution from a metal stud machined on the metallic adherent surface using RFSSW. The conceptual design process started with the development of a methodology and the identification of performance indicators based on the list of requirements. This was followed by a simulation program, which offered answers regarding the required maximum bearing load and overall stiffness of the joint, but showed some limitations in terms of predictive modelling of the post-peak load behaviour. A complete experimental program, performed with two sizes of the bonding area, clearly demonstrated several advantages in the joint performance compared to the reference adhesively bonded joints and provided a fundamental understanding of the joint behaviour. The novel joint exhibited a greater maximum load and, more importantly, greater fracture toughness than the reference joint. The experimental program demonstrated that the linear behaviour of the joints is controlled by the stiffness and strength of the bonding area; however, the novel joint offers a significant post-peak residual strength and capacity for damage tolerance. Importantly, the novel design offers features known for classic mechanical fasteners, but without any added material and aesthetic penalties. Consequently, the industrial impact is expected to extend beyond naval applications to other weight-sensitive structures. Future work should focus on fatigue testing of the joint and its performance in a marine environment.

## Figures and Tables

**Table 1:** Geometry of the novel joint specimens.

Specimen Type	Edge Distance to Hole Diameter Ratio (e/d)	Width to Hole Diameter Ratio (w/d)	Bonding Length
MSJ-L24	3	6	24 mm
MSJ-L48	6	6	48 mm

## Data Availability

The data presented in this study are available on request from the corresponding author due to legal reasons.

## Appendix A

A comparison of the maximum loads obtained in the tests with the reference joints, L48-RIJ, and the new joints L48-MSJ is shown in Figure A1, while the complete set of data measured in the experiment with the L24-MSJ and L48-MSJ specimens are listed in Table A1 and Table A2, respectively, along with the mean and standard deviation.

**Table A1:** Maximum load and displacement measured in the L24-MSJ tests, with the mean and standard deviation.

Specimen	Maximum Load, kN	Displacement, mm
L24-MSJ-01	5.36	0.34
L24-MSJ-02	5.06	0.33
L24-MSJ-03	5.3	0.32
L24-MSJ-04	5.99	0.37
L24-MSJ-05	4.96	0.29
L24-MSJ-06	5.64	0.36
L24-MSJ-07	6.4	0.44
Mean	5.53	0.35
Standard Deviation	0.48	0.05

**Table A2:** Maximum load and displacement measured in the L48-MSJ tests with mean and standard deviation values.

Specimen	Maximum Load, kN	Displacement, mm
L48-MSJ-01	6.22	0.42
L48-MSJ-02	5.33	0.43
L48-MSJ-03	6.71	0.45
L48-MSJ-04	4.62	0.27
L48-MSJ-05	5.37	0.32
L48-MSJ-06	3.80	0.30
Mean	5.34	0.37
Standard Deviation	1.05	0.08
